# Screening target genes for the treatment of PCOS via analysis of single-cell sequencing data

**DOI:** 10.1080/07853890.2022.2136401

**Published:** 2022-10-26

**Authors:** Zhenzhen Lu, Chunyan Chen, Ying Gao, Yanhui Li, Xiaojie Zhao, Hanke Zhang, Qiongqiong Wei, Xinliu Zeng, Yajie Li, Min Wan

**Affiliations:** Department of Gynecology and Obstetrics, Union Hospital, Tongji Medical College, Huazhong University of Science and Technology, Wuhan, China

**Keywords:** Differentially expressed genes, Insulin resistance, Polycystic ovary syndrome, Biomarkers, Gene therapy

## Abstract

**Background:**

Polycystic ovary syndrome (PCOS) is a condition of the female reproductive system and it remains imperative to identify target genes responsible for its pathogenesis and develop therapeutic drugs capable of effectively treating it.

**Methods:**

We performed primary screening, staging, functional analysis as well as screening of target genes and therapeutic drugs based on single cell sequencing data of 34 oocytes from the GEO database.

**Results:**

Oxidative phosphorylation played a pivotal role in the development of oocytes, insulin resistance and ovulation disorders. At the cellular level, GV and MI phases were particularly critical for the biology of pregnancy. We screened *PGR*, *SIRT1* and *ADAMTS1* as hub differentially expressed genes (DEGs) and found relevant drugs using the Drug-Gene Interaction Database. In clinical study, oral contraceptives and insulin sensitisers were found to be effective in the treatment of PCOS.

**Conclusion:**

*PGR*, *SIRT1* and *ADAMTS1* were found to be down-regulated in oocytes, ovulation and female pregnancy. These 3 genes are likely biomarkers important in the treatment of PCOS. Insulin sensitiser in combination with oral contraceptive administration were found to significantly improve PCOS.

**Key messages**
- Our study used a new bioinformatics approach to find target genes for the treatment of PCOS.
- Our study sought to identify target genes that affect human oocyte quality by analysing single-cell sequencing data from oocytes.
- We testified to our data by analysing a subset of clinical data.

## 1. Introduction

Polycystic ovary syndrome (PCOS) is a female endocrine disorder affecting 5–10% of reproductive age patients [1]. The Rotterdam diagnostic criteria is generally utilized in the diagnosis of this condition [2] and ovulatory dysfunction, hyperandrogenism and polycystic ovarian morphology (PCOM) are typical manifestations [3]. Insulin resistance (IR) is an especially prominent characteristic of PCOS [4] and about 75% of patients suffer impaired insulin sensitivity [5]. Infertility in the setting of PCOS accounts for a large percentage of anovulatory infertility [6]. Despite significant research efforts the underlying mechanisms of abnormal follicular development and anovulation remains unclear [7]. However, evidence indicates that environmental, developmental, genetic and epigenetic factors are all important in the pathogenesis of PCOS [8,9]. Furthermore, hyperandrogenism is understood to play a major role in metabolic disorders [3,10]. As elevated serum testosterone and luteinizing hormone (LH) are typical biochemical features of PCOS [11], oligoovulation, androgen excess and IR are accepted treatment targets [12]. Therapeutic strategies include metformin administration and weight management [13,14], both of which can reduce IR but can’t reverse IR [15].

Single-cell RNA sequencing (scRNA-seq) [16] measures gene expression at the cellular level and yields higher resolution data as compared to bulk RNA-sequencing in addition to improving the understanding of cellular activity, disease progression and treatment response [17,18]. Here, we used scRNA-seq (GEO, https://www.earthobservations.org/index.php; accession number PRJNA600740) to study 34 oocytes obtained from both healthy individuals and PCOS patients to identify important processes in the pathogenesis of PCOS and screened drugs for treatment potential. Alterations in endocrinology and glucose metabolism were evaluated for the purposes of clinical validation of treatment.

## 2. Methods

### 2.1. Data acquisition

The data of 20 oocytes from PCOS patients and of 14 from healthy individuals were acquired from the Intergovernmental Group on Earth Observations (GEO, https://www.earthobservations.org/index.php; accession number PRJNA600740). Data were processed using RStudio software and R v. 4.0.4, platform x86_64-w64-mingw32/x64 (64-bit).

### 2.2. Data filtering, dimensional reduction, and pseudotime analysis

As only 34 oocytes were studied, data were not filtered according to gene and cell quantity or mitochondrial DNA percentage. Gene quantity and mitochondrial DNA percentage were noted, however, and the top 10 genes with highly variable expression among cells were identified. Available dimensions above the dotted line are detailed using a JackStraw plot. The DimHeatmap function based on individual principal components was applied, and cells and genes were sorted based on principal component scores. Nonlinear dimensionality reduction using t-SNE clustering was also performed. The FindAllMarkers function was applied to identify markers significantly expressed among clusters, and t-SNE plots of the top 4 markers in the cluster were constructed and total mRNAs of the two groups were shown. Pseudotime analysis differentiated the relationship among subpopulations and revealed functional alterations during the differentiation process.

### 2.3. Cellphonedb, GO and signalling pathway analyses of oocytes

CellPhoneDB is a publicly available repository of curated receptors, ligands and their interactions [19]. EdgeR packages were used to identify differentially expressed genes (DEGs), P value <0.05 was considered as the differential expression thresholds, and a value of logFC >1.5 was considered as signifying up regulation while logFC <−1.5 considered as signifying down regulation; P value >0.05 were considered as signifying stability. PCA plots, Volcano plots and Heatmaps were constructed and DEGs with P values of <0.00001 & abs (DEG$logFC) ≥3 were labelled. Up regulated DEGs were marked red, down regulated DEGs were marked blue while stable DEGs were marked. Ggplot2, clusterProfiler and org.Hs.eg.db packages were utilised to constructed bar graphs of gene ontology (GO) functions [20,21], and KEGG pathways of studied oocytes.

### 2.4. Copykat, GSEA GO and KEGG pathway analyses of aneuploid oocytes

Cells exhibiting aneuploidy alterations were analysed using CopyKAT analysis and t-SNE plots were constructed. Screening conditions for DEG evaluation as well as volcano plots construction were as detailed above. Gene Set Enrichment Analysis (GSEA) was applied for GO and KEGG pathway study of aneuploid oocytes and Biological processes (BP) cnetplots and gseaplot2 analyses of GV, MI, and MII phases was used. Protein-Protein Interaction (PPI) networks [22,23] of aneuploid oocytes were constructed using STRING (v. 11.0), with a combined score of >0.4 (medium confidence); DEGs were selected with |logFC| of >3 using Cytoscape software (v. 3.7.1). MCODE (v. 1.6.1) was used to identify modules of greatest significance; criteria for selection were as follows: degree cut-off = 2, node score cut-off = 0.2, max depth = 100 and k-score = 2.

### 2.5. Meaningful biological process, target DEG and relevant drug screening analyses

Circular cnetplots and gseaplot2 of significant BPs in all 3 phrases were constructed by using RStudio. Venn diagrams of DEGs were constructed using Bioinformatics & Evolutionary Genomics (http://bioinformatics.psb.ugent.be/webtools/Venn/) and target DEGs were identified. The Drug-Gene Interaction Database (DGIdb, http://www.dgidb.org), a website that consolidates disparate data sources detailing drug-gene interactions [24], was utilised to identify drug-gene interactions.

### 2.6. Validation of clinical drug therapy in patients with PCOS

**Figure 1.**
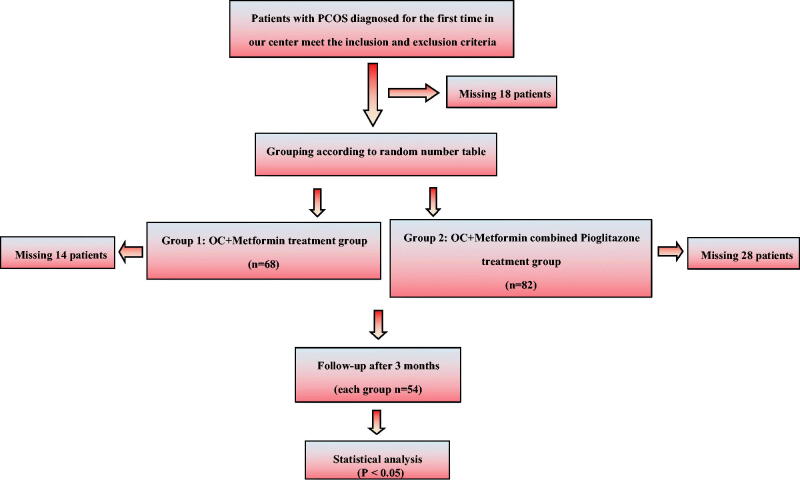
Clinical research flow chart.

A total of 168 patients were selected according to inclusion and exclusion criteria (We declared that our research was guided by the principles of the Declaration of Helsinki, and all treatments received verbal consent from patients, this clinical study was approved by the Ethics Committee of Union Hospital, Tongji Medical College, Huazhong University of Science and Technology (ethical approval number for human research: 0241-01) ), after missing 18 patients in the course of the study, there remained 150 patients. During the follow-up group treatment, OC + Metformin group missed 14 patients, OC + Metformin combined Pioglitazone missed 28 patients, so 108 were eventually tested on their 2nd-5th days of menstruation. Endocrine and metabolic data from January 2017 to December 2017 were compiled (Figure 1). Patients in group 1 were administered oral contraceptives (OC; Diane-35) starting on day 5 of menstruation along with metformin 500 mg bid/d for 3 consecutive menstrual cycles. Patients in group 2 were administered Diane-35 starting on day 5 of menstruation along with metformin 500 mg bid/d and pioglitazone 15 mg bid/d for 3 consecutive menstrual cycles. After 3 months, endocrine and glucose metabolism data were statistically analysed using SPSS v.26.0; *p* < 0.05 was considered statistically significant.

## 3. Results

### 3.1. Data filtering, dimensional reduction and pseudotime analysis

**Figure 2.**
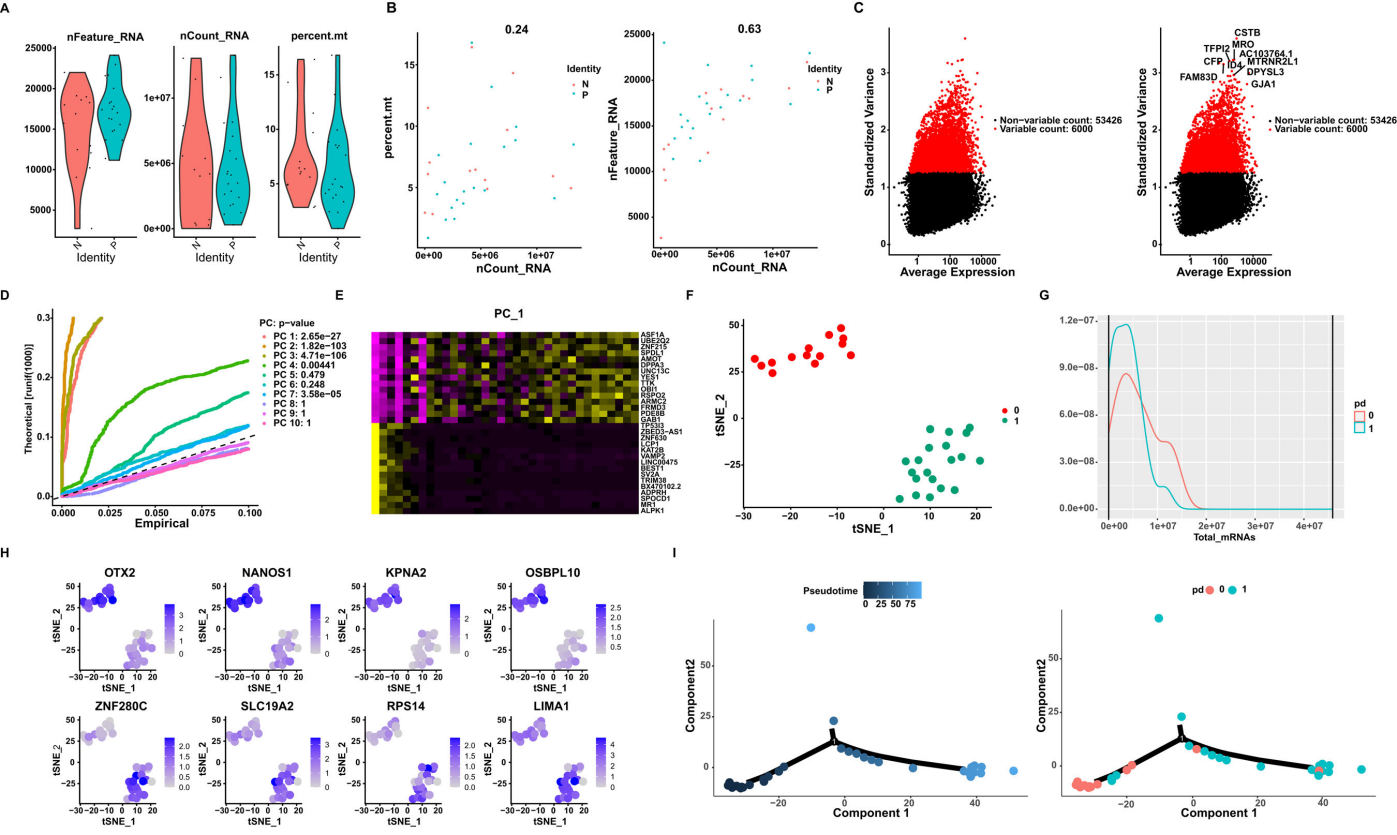
A. VlnPlot. B. FeatureScatter Plot. C. The top 10 VariableFeature Plot. D. JackStraw Plot. E. PC_1 heatmap. F. TSNE Plots. G Total mRNAs of the two groups. H. FeaturePlot of top 4 markers. I. Proposed timing analysis plots.

Single cell sequencing data were filtered and discriminated for characteristics. Violin diagram construction revealed gene and cell quantities and mitochondrial gene percentages (Figure 2(A)). Gene quantities and mitochondrial gene percentages in relation to cell quantity were shown in Figure 2(B). The 10 most highly variable genes were shown in Figure 2(C). JackStraw plotting was used to detail available data dimensions of the data as shown in Figure 2(D). A heatmap of PC1 was shown in Figure 2(E), it represents the new variables obtained by transforming the variables in the original data. Nonlinear dimensionality reduction clustering *via* t-SNE was shown in Figure 2(F) and mRNAs of the two groups Figure 2(G), the top 4 DEGs in each cluster were shown in Figure 2(H). Pseudotime analysis plots and total mRNA content of the 2 clusters were shown in Figure 2(I).

### 3.2. Cellphonedb, GO and signalling pathway analyses of oocytes

**Figure 3.**
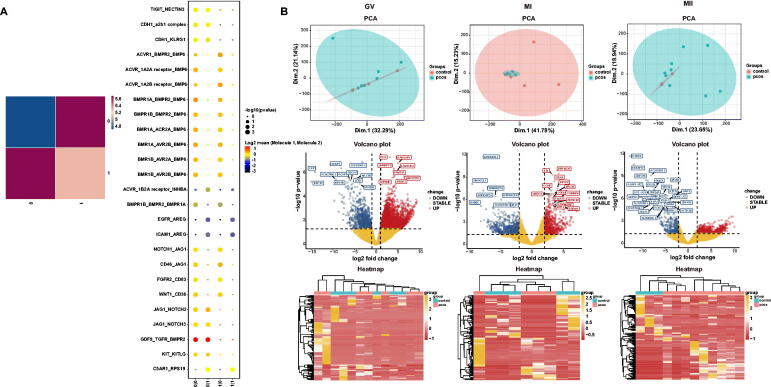
A. Heatmap of logCount and CellphoneDB. B. PCA plots, volcano plots, heatmaps of GV, MI and MII phases.

**Figure 4.**
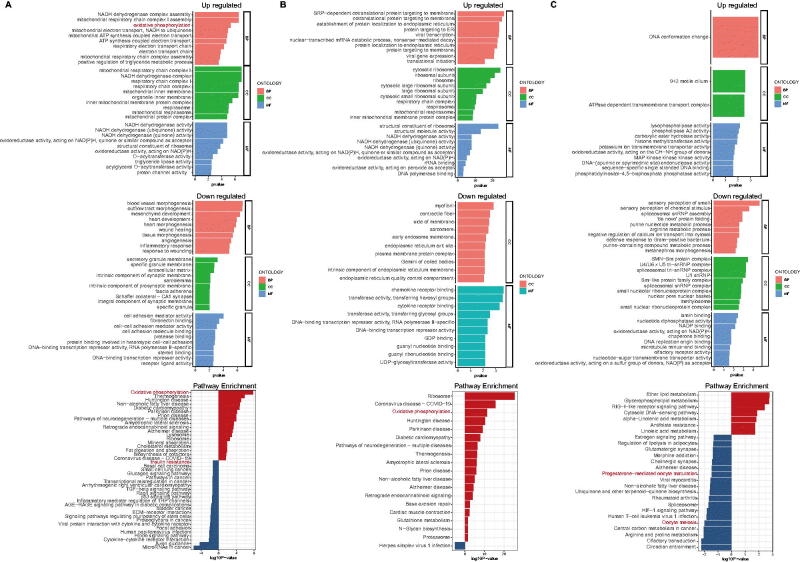
A. GO functions and signalling pathways in GV phase. B. GO functions and signalling pathways in MI phase. C. GO functions and signalling pathways in MII phases.

CellphoneDB analysis of ligand/receptor expression using scRNA-seq data revealed inter-oocyte interactions and were shown in Figure 3(A). PCA, volcano and heatmap plots (Figure 3(B)), up and down of GO functions, and signalling pathways of GV, MI and MII oocyte phase (Figure 4). In the three phases, oxidative phosphorylation was found to be important both in GO and oocyte pathway during the development of oocyte. In phase GV, IR and glucagon signalling were down regulated and in MII phase, progesterone-mediated oocyte maturation and oocyte meiosis were down regulated.

### 3.3. Copykat, GSEA GO and KEGG pathway analyses of aneuploid oocytes

**Figure 5.**
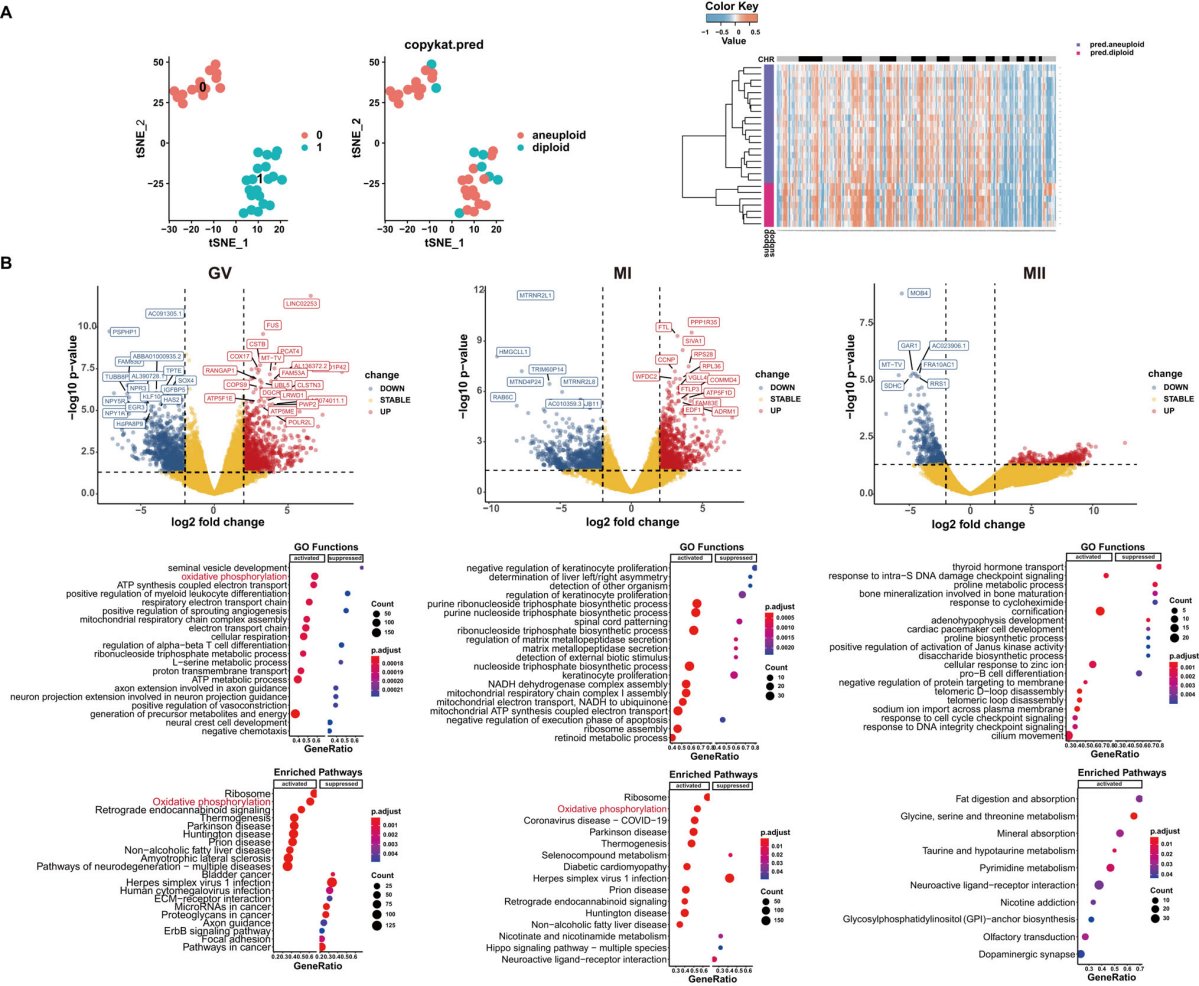
A. t-SNE plots and CopyKAT plot of aneuploid oocytes. B. Volcano plots, GSEA GO and KEGG enriched bubble plots of GV, MI and MII phases in aneuploid cells.

**Figure 6.**
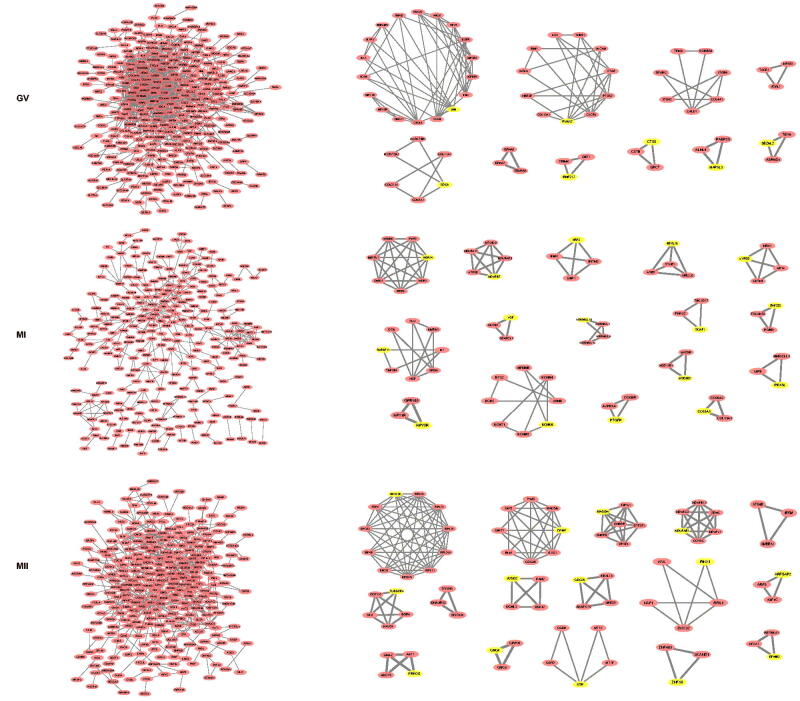
PPI networks of GV, MI and MII in aneuploid cell.

Data detailing the t-SNE of clusters 0 and 1, the number 0 represented normal oocytes and the number 1 represented oocytes with PCOS, aneuploid and diploid oocytes, while heatmaps of pred.aneuploid and pred.diploid data were shown in Figure 5(A). In Figure 5(B), Volcano plots of GV, MI and MII phases were shown. Oxidative phosphorylation was determined the most important factor in all the three phases of oogenesis. Biological processes related to ovulation disorders, female pregnancy, and meiosis were shown greater detail in the three phases. The PPI networks of DEGs from all phases of oocytes were constructed (Figure 6), three phases of the MCODE modules were constructed, and seed DEGs were highlighted yellow.

### 3.4. Meaningful BP, target DEGs, and relevant drug analyses

**Figure 7.**
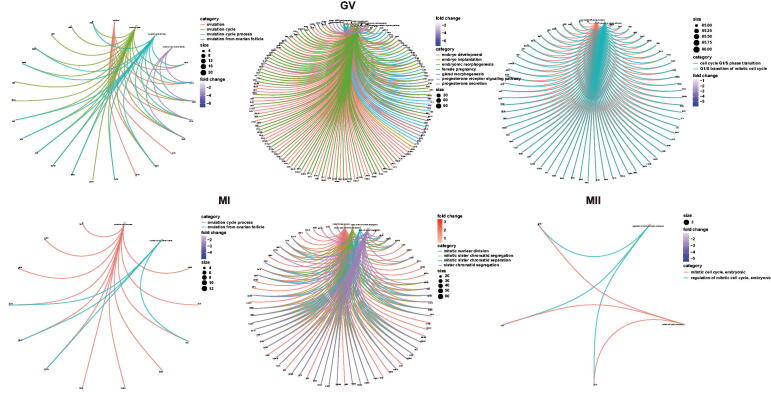
Cnetplots of significant biological process from GV, MI, and MII phases in aneuploid cells.

**Figure 8.**
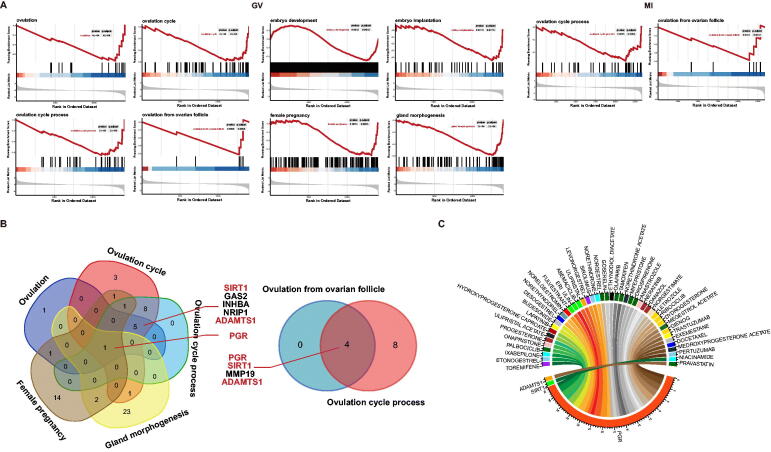
A. GSEA plots from GV, MI phases in aneuploid cells. B. Venn diagrams of significant biological processes in GV and MI phases. C. Drug-Gene Interaction Chord Diagram of PGR, SIRT1, and ADAMTS1.

Cnetplots of significant BP from GV to MII phases were shown in Figure 7, and GSEA plots from GV to MI phases in aneuploid oocytes were shown in Figure 8(A). In the GV phase, BPs associated with ovulation were found to be significantly down regulated, including ovulation, ovulation cycle, ovulation cycle process and ovulation from ovarian follicle. Those BPs associated with pregnancy were found to be down regulated, including those associated with embryo development and embryo implantation, female pregnancy, gland morphogenesis and progesterone secretion. Meiosis-associated BPs were found to be down regulated, including those associated with meiotic G1/S and G1/S transitions. Throughout the MI phase, processes associated with the ovulation cycle were found to be down regulated, although BPs related to meiosis were up regulated, including meiotic nuclear division and mitotic sister chromatid segregation. In the MII phase, In MII phase, mitotic cell cycle, and regulation of mitotic cell cycle were also down regulated, but absence of BP associated with ovulation in MII phase. Because the main BPs associated with obstacles to ovulation were noted in GV and MI phases, Venn diagrams were utilised to identify common DEGs involved in BPs relevant to ovulation and pregnancy (Figure 8(B)). Notably, *PGR* (Progesterone Receptor), *SIRT1* (Sirtuin 1) and *ADAMTS1* (A disintegrin and metalloproteinase with thrombospondin motifs 1) were found to be involved in ovulation; *PGR* also was noted to participate in pregnancy and gland morphogenesis (Table 1). Finally, PGR, *SIRT1* and *ADAMTS1* were input into DGIdb to identify potential drugs; PGR was found to be potentially targeted by 42 drugs, and *SIRT1* and *ADAMTS1* each by 1 drugs (Figure 8(C), Table 2).

**Table 1.** DEGs of significant biological processes in GV and MI phases.

Phase	Biological Processes	Gene symbol
GV	ovulation	*GAS2, SIRT1, NRIP1, PGR, ADAMTS1, TNFAIP6, INHBA, PTGS2*
	ovulation cycle	*PDGFRA, ADNP, MAP2K6, PLEKHA1, GAS2, AMH, BMPR1B, CASP3, SIRT1, NRIP1, PGR, ADAMTS1, OXTR, AXL, INHBA, PAM, PTX3, HAS2, EGFR, NPY5R*
	ovulation cycle process	*PDGFRA, MAP2K6, PLEKHA1, GAS2, AMH, BMPR1B, CASP3, SIRT1, NRIP1, PGR, ADAMTS1, INHBA, PAM, PTX3, NPY5R*
	female pregnancy	*ACSL4, AR, STC1, CRHBP, PGR, OXTR, PTHLH, VEGFA, ITGA5, IGFBP7, PRDM1, SLC38A2, TRO, PAPPA, PAM, ITGA2, IGFBP5, GJA1, STC2, PTGS2*
	gland morphogenesis	*FGF7, PROX1, NTN4, GLI3, LAMA1, FGFR2, SFRP1, NFIB, AR, TWSG1, EPHA2, AREG, PGR, MSN, WNT5A, CAV1, NOTCH2, SEMA3A, SOX9, TNFAIP3*
MI	ovulation cycle process	*SLIT3, CASP2, MMP19, MAP2K6, ZNF830, PAM, SIRT1, ADAMTS1, PGR, NPY5R, EREG, LHCGR*
	ovulation from ovarian follicle	*MMP19, SIRT1, ADAMTS1, PGR*

**Table 2.** Drugs targeting *PGR*, *SIRT1* and *ADAMTS1* from DGIdb.

Gene	Drug	Interaction Type & Directionality	PMIDs	Score
***PGR***	ULIPRISTAL	modulator	23437846	5.7
	LEVONORGESTREL	agonist, modulator, binder	17484506 17531625 17077229 16157482 11752352 16084894	4.18
	DYDROGESTERONE	agonist	6827166 436795 11752352 16045524 8486204 15128769	4.18
	DESOGESTREL	agonist	6645495 3139361 11041225 11752352 7750284 10601100	2.85
	DROSPIRENONE	agonist	7625729 8922878 16291771 17000933 16157482 15493951 11024226	2.85
	NORELGESTROMIN	agonist	17107221 1324557 19962254 7750291 8384965	2.66
	ETONOGESTREL	agonist	15063480 11041225 11752352 10601100	2.28
	NORGESTIMATE	agonist	17107221 1324557 11752352 7750291 8384965	2.05
	NORETHINDRONE	agonist	15063480 15261304 10494488 15189034 7711211 11752352 16084894	1.52
	ETHYNODIOL DIACETATE	agonist	166800 858280 11752352	1.52
	ONAPRISTONE	antagonist	None found	1.14
	ULIPRISTAL ACETATE	modulator	None found	1.14
	DIENOGEST	agonist	18061638	1.14
	NORGESTREL	agonist, binder	11521119	0.76
	NORETHYNODREL	agonist	None found	0.57
	NORETHINDRONE ACETATE	agonist	None found	0.57
	MIFEPRISTONE	agonist, antagonist	10699595 10682471 10374120 10806733 11783365 11752352	0.57
	MEDROXYPROGESTERONE ACETATE	agonist	12846422	0.41
	DANAZOL	agonist	17636649 2404115 8000225 18061638	0.36
	TOREMIFENE	n/a	2147123	0.33
	ERIBULIN	n/a	None found	0.33
	GOSERELIN	n/a	None found	0.33
	HYDROXYPROGESTERONE CAPROATE	agonist	None found	0.28
	MEGESTROL ACETATE	agonist	None found	0.28
	PERTUZUMAB	n/a	None found	0.28
	ANASTROZOLE	n/a	None found	0.24
	LETROZOLE	n/a	None found	0.23
	PROGESTERONE	agonist	17109827 9506743 21315613 17138644 17013809 17015480 17169175 30013421	0.22
	NERATINIB	n/a	None found	0.21
	FULVESTRANT	n/a	None found	0.16
	EXEMESTANE	n/a	None found	0.16
	RIBOCICLIB	n/a	None found	0.14
	IXABEPILONE	n/a	None found	0.11
	BUDESONIDE	agonist	None found	0.09
	TAMOXIFEN	n/a	3793272	0.09
	TRASTUZUMAB	n/a	None found	0.09
	LAPATINIB	n/a	None found	0.08
	ABEMACICLIB	n/a	None found	0.08
	OLAPARIB	n/a	None found	0.08
	SIROLIMUS	n/a	1284044	0.04
	DOCETAXEL	n/a	None found	0.03
	PALBOCICLIB	n/a	None found	0.02
***SIRT1***	NIACINAMIDE	n/a	25884115	1.64
***ADAMTS1***	PRAVASTATIN	n/a	18174457	3.65

### 3.5. Efficacy of insulin sensitisers in the treatment of PCOS

In the comparison of the underlying conditions, both groups significantly differed in terms of weight and BMI reduction. Androgen and serum LH levels were found to be significantly lower in both groups. The differences among group 1 subject glucose indices were not statistically significant, while those in group 2 were found to have significantly decreased HOMA-IR, glycosylated haemoglobin level and CRP, suggesting that a combination of insulin sensitisers produced superior results in terms of improving glucose metabolism (Table 3).

**Table 3.** Comparison of patient groups before and after treatment in terms of general condition, endocrine parameters and glucolipid metabolism.

Terms		group 1 (*n* = 54)		T or Z	P*	group 2 (*n* = 54)		T or Z	P*	△group1	△group1	T or Z	P*
		Pre-treatment	Post-treatment			Pre-treatment	Post-treatment						
Basic conditions	Age	26				25							
	Height (cm)	161				162							
	Weight (kg)	64.2	61.7	−5.396	<0.001	63.9	61.2	−4.007	<0.001	−3.15 ± 3.11	−2.133 ± 3.64	1.572	0.119
	BMI (kg/m^2^)	25.35	24.55	−4.591	<0.001	24.65	23.65	−4.158	<0.001	−1.25	−0.85	−0.732	0.464
	Waist-hip ratio	0.88	0.88	−2.377	0.017	0.88	0.87	−0.867	0.386	−0.01	−0.01	−0.903	0.367
	Visceral fat area	115.23 ± 35.17	109.09 ± 30.97	2.771	0.008	109.44 ± 30.48	105.78 ± 28.72	1.832	0.073	−4.70	−3.15	−1.241	0.215
	Body fat percentage	25.89 ± 5.44	34.75 ± 5.89	0.147	0.147	35.00 ± 5.38	34.63 ± 4.90	0.618	0.540	−0.85	−0.10	−1.530	0.126
Endocrine changes	FSH	5.89	5.97	−1.124	0.261	5.71	6.02	−1.881	0.060	0.32	0.27	−0.461	0.645
	LH	9.78	7.47	−2.722	0.006	12.99	7.55	−3.414	<0.001	−1.98	−4.79	−1.220	0.223
	LH/FSH	1.82	1.16	−4.077	<0.001	2.08	1.18	−4.275	<0.001	−0.65	−0.78	−1.238	0.216
	T	1.50 ± 0.59	1.27 ± 0.53	2.944	0.005	1.58 ± 0.58	1.56	−0.194	0.847	−0.27	−0.13	−2.089	0.037
	DHEA	32.14	23.21	−2.614	0.009	29.73	25.17	−2.174	0.030	487 ± 13.26	−5.79 ± 17.17	−0.279	0.781
	DHT	9.74	6.83	−4.244	<0.001	9.18	7.22	−2.587	0.010	−2.29 ± 3.47	-l.34 ± 3.43	1.432	0.155
	FT	9.92	5.28	−4.490	<0.001	7.93	5.74	−5.106	<0.001	−3.14	−2.79	−0.012	0.990
	El	267.4	193.65	−4.736	<0.001	235.85	174.85	−2.915	0.004	•89.62	−53.45)	−2.363	0.018
	E2	136.25	104.0	−2.860	<0.001	128.05	132.35	−1.402	0.161	−44.11	−22.03	−0.826	0.409
	SHBG	36.02	147.70	•5.739	<0.001	42.77	200	−5.704	<0.001	91.24	128.61	−2.529	0.011
	FAI	4.04	0.82	−5.954	<0.001	3.31	1.01	−5.928	<0.001	−3.44	−2.52	−0.568	0.570
Glucose metabolism	CRP	1.21	1.51	−0.198	0.843	0.83	0.55	−2.700	0.007	0.09	−0.29	−1.865	0.062
	FPG	5.40	5.05	−1.519	0.129	5.11 ± 0.57	5.00 ± 0.65	−2.470	0.014	−0.25	−0.30	−0.188	0.198
	FINS	13.99	14.42	−0.495	0.621	14.31	11.31	−1.890	0.059	−0.54	−1.81	−0.919	0.358
	IUCA	276.62	325.70	−1.985	0.047	265.05	269.84	−0.564	0.573	33.06	−4.02	−1.674	0.094
	HOMA-IR	3.74	3.21	−0.254	0.799	3.20	2.48	−2.174	0.030	−0.34	−0.53	−1.115	0.265
	HbAlc	5.15	5.10	−1.072	0.284	5.1610.28	5.1610.28	−2.540	0.011	−0.05	−0.10	−0.754	0.451
	0.5G1U	8.90	8.95	−0.164	0.870	9.18 ± 1.43	8.70 ± 1.23	2.191	0.033	0.45	−0.40	−1.288	0.198
	IhGlu	9.35	9.35	−0.818	0.413	8.97 ± 2.67	8.90 ± 2.09	0.180	0.858	−0.20	−0.05	−0.688	0.491
	2hGlu	7.20	7.30	−0.108	0.914	6.65	7.60	−1.637	0.102	0.00	0.30	−1.202	0.230
	3hGlu	4.80	4.90	.0.173	0.863	4.66 ± 1.07	4.6811.45	−0.061	0.952	0.10	0.15	−0.212	0.832
	0.5hIns	109.45	116.97	−1.795	0.073	102.63	96.59	−0.555	0.579	15.55 ± 66. 19	−8.94 ± 65.79	−1.929	0.056
	Ihlns	117.19	149.47	−2.553	0.011	114.02	125.60	−0.047	0.962	19.10	−10.08	−1.816	0.069
	2hlns	116.10	128.55	−1.649	0.099	99.14	97.75	−0.504	0.614	16.01 ± 66. 35	−8.94 ± 65.79	−1.658	0.100
	3hlns	44.63	35.49	−0.779	0.436	31.18	26.22	−2.001	0.045	−7.94	−3.75	−0.802	0.423
	HOMA•B	185.57	190.14	−0.883	0.377	143.77	138.16	−0.022	0.983	15.80	4.98	−0.752	0.452

## 4. Discussion

Here, GO and KEGG functions of 34 oocytes at GV, MI and MII stages, as well as of aneuploid oocytes, revealed that the pathway responsible for IR mainly functioned in the GV phase, while processes associated with ovulation disorders were mainly down regulated in GV and MI phases. Analysis of DEGs involved in the ovulation process during GV and MI phases revealed *PGR*, *SIRT1* and *ADAMTS1* to be hub DEGs, and all to down regulated in the setting of PCOS. Diseases associated with *PGR* mainly include progesterone resistance and myoma formation, with the responsible cellular process being oocyte meiosis. *PGR* plays a central part in reproductive events associated with the establishment and maintenance of pregnancy, and encodes a member of the steroid receptor superfamily, its expression was previously found to be up regulated in the *PCOS* endometrium [25–27], while in our study, it was found to be down regulated in oocytes. *SIRT1* was associated with ageing, cellular senescence and the p53 pathway, the level of serum *SIRT1* was to be higher in PCOS patients as compared to controls [28], but the expression of SIRT1 was significantly lower in ovarian tissues as compared to controls in the setting of PCOS, and it was consistent with our findings. Exenatide has been reported to be of therapeutic value for PCOS by its up regulation of *SITR1* expression [29,30]. *ADAMTS1* was reported relevant to premature ovarian failure and menopause and to be significantly increased in granulosa cells in PCOS [31]. *ADAMTS1* expression was found to exhibit a positive correlation with oocyte fertilisation rate [32] and more mature oocytes, transplantable embryos and better-quality embryos [33]; however, we found it to be significantly down regulated in our analysis. *via* the screening of drugs relevant to these 3 DEGs, we found that most therapeutic drugs targeted *PGR* and the majority of these were progesterone drugs, exerting both progestogenic and anti-androgenic effects. In clinical treatment, the most common strategy targeting IR includes the use of insulin sensitiser drugs, particularly metformin, which produces similar effects to lifestyle interventions such as a decreased body weight, but is superior in terms of decreasing androgen concentrations, and it was consistent with our clinical study. Furthermore, the combination of metformin and OC administration likely prevents any deterioration in metabolic function.

Oligoovulation and anovulation are the primary aetiologies for female infertility in PCOS, resulting in menstrual dysfunction and endometrial hyperplasia, thereby increasing the risk of endometrial cancer [34]. Administration of OC has been traditionally used for endometrial protection and attenuation of hyperandrogenism [35] and the use of a levonorgestrel-releasing intrauterine device, these were essentially consistent with screening drugs in Table 2. Metformin was also reported to assist with the prevention of ovarian hyperstimulation syndrome in the setting of *in vitro* fertilisation [36] and improved ovulation and pregnancy rates [8]. Here, we validated Diane-35 tablets which contained cyproterone acetate (2 mg) and ethinylestradiol (0.035 mg). Cyproterone acetate, previously screened for associating with foetal growth restriction, exerts both progestogenic and anti-androgenic effects. Metformin and pioglitazone are insulin sensitisers that improve IR. Diane-35 combined with metformin was found to significantly increase *SIRT1* expression in rat PCOS ovarian tissues [30], and combination therapy resulted in decreased body weights, levels of luteinizing hormone and testosterone and IR[30]. Here, the combination of Diane-35 with metformin was found to significantly reduce weight and BMI in both groups as compared to pre-treatment. No statistical difference among group 1, using one insulin sensitiser, and group 2, using two insulin sensitisers, was noted. In addition, serum androgen and luteinizing hormone levels in both groups decreased significantly compared to pre-treatment, with no significant difference noted among groups. This phenomenon likely manifested due to cyproterone acetate reducing androgen levels and inhibiting hypothalamic GnRHa, thereby leading to a decrease in serum luteinizing hormone levels. Ultimately, a combination of insulin sensitisers is likely more effective in improving glucose metabolism.

## 5. Conclusion

The pathogenesis of PCOS and the development of ovulation disorders in this condition remains unclear. Metformin, however, is known to improve IR and ovulation. In the clinical setting, clomiphene citrate and letrozole are considered to be the first-line agents for ovulation induction [36]. Metformin reduces the risk of ovarian hyperstimulation syndrome, which together with ovarian laparoscopic surgery are used as second-line treatment [36]. Although the *PGR* progesterone agonist drugs have been studied in greater detail, methods of *SIRT1* and *ADAMTS1* modulation require further investigation. Besides it is difficult to collect oocyte from PCOS and control patients to do further validation, which is a limitation of our research, so it need to be validated by further subsequent studies.

## Data Availability

All datasets involved in the article are available in the GEO database (GEO, https://www.earthobservations.org/index.php; accession number PRJNA600740).
